# Comparison of da Vinci Robotic Cholecystectomy and Laparoscopic Cholecystectomy: A Systematic Review and Meta-Analysis of Postoperative Outcomes and Cost-Effectiveness

**DOI:** 10.7759/cureus.73767

**Published:** 2024-11-15

**Authors:** Poonam Tawde, Noel John, Seynab Farah, Mehdi D, David Stuart

**Affiliations:** 1 Department of Surgery, Avalon University School of Medicine, Willemstad, CUW; 2 Department of Physical Therapy, University of Nottingham, Nottingham, GBR; 3 Department of Emergency Medicine, Avalon University School of Medicine, Willemstad, CUW; 4 Department of Internal Medicine, Avalon University School of Medicine, Willemstad, CUW; 5 Department of General Surgery, Beckley Appalachian Regional Hospital, Beckley, USA

**Keywords:** comparative trials, cost-effectiveness, da vinci surgical system, laparoscopic cholecystectomy, minimally invasive surgery, postoperative complications, postoperative outcomes, robotic cholecystectomy, surgical outcomes, systematic review

## Abstract

Robotic cholecystectomy (RC) using the da Vinci surgical system has been introduced as a potential alternative to the gold standard laparoscopic cholecystectomy (LC) for gallbladder removal. This systematic review aims to evaluate and compare the postoperative outcomes (operative time, bile leak, and postoperative complications) and cost-effectiveness between da Vinci RC and LC. A comprehensive search of electronic databases, including EMBASE, MEDLINE, Cochrane Library, and PubMed, used Medical Subject Headings terms and Boolean operators to identify relevant studies. Comparative trials assessing postoperative outcomes and costs between RC and LC were included. Data were extracted independently by researchers and analyzed using the RevMan 5.4 software (The Cochrane Collaboration, London, UK). The analysis included six studies with a total of 1,013 patients, comprising three randomized controlled trials (RCTs) and three cohorts conducted across Switzerland, Taiwan, the USA, and Italy. Results showed that LC had a significantly shorter operation duration than RC (standardized mean difference: 0.27; 95% CI, 0.05-0.49; p = 0.01). No significant differences were observed between RC and LC in terms of bile leak rates (odds ratio, 0.37; 95% CI, 0.06-2.21; p = 0.27) or postoperative complications (odds ratio, 0.50; 95% CI, 0.18-1.37; p = 0.18). Cost analysis revealed that RC was more cost-effective than LC (standardized mean difference, 3.16; 95% CI, 0.39-5.93; p = 0.03), with substantial heterogeneity noted among the studies. The findings suggest that RC does not provide significant clinical advantages over LC in postoperative outcomes and incurs higher costs. Due to the heterogeneity and the limited number of RCTs included, a major multicenter RCT is recommended to validate these results further. In conclusion, LC remains the preferred approach due to its shorter operative time and cost-effectiveness, with no significant differences in clinical outcomes compared to da Vinci RC. Further research with larger, multicenter trials is warranted to confirm these findings and guide clinical decision-making.

## Introduction and background

Robotic cholecystectomy (RC), performed using the da Vinci surgical system, has emerged as a potential alternative to the gold-standard laparoscopic cholecystectomy (LC). LC has been widely adopted for its minimally invasive approach, shorter hospital stays, and lower complication rates than open cholecystectomy [1]. The introduction of robotic systems, particularly the da Vinci platform, aims to overcome the limitations of conventional laparoscopy by offering enhanced precision, dexterity, and visualization [2].

Robotic surgery has shown promising outcomes across various surgical fields, including gynecology and urology, by providing surgeons with improved ergonomics and reducing fatigue [3]. In RC, these advantages translate into more precise surgical movements and the ability to perform complex tasks with greater ease. Proposed benefits of RC over LC include reduced blood loss, decreased postoperative pain, and a lower risk of bile duct injuries due to enhanced visualization and control [4,5]. However, the adoption of RC is often limited by higher costs associated with robotic systems, longer setup times, and specialized training requirements [3].

The comparative effectiveness of RC and LC in cholecystectomy has been debated in the literature, with mixed findings regarding their benefits. Some studies suggest that RC may offer advantages, such as reduced intraoperative blood loss and decreased postoperative pain, potentially leading to a more favorable recovery experience for patients [4,5]. However, other research has found no significant differences in key clinical outcomes, such as complication rates and length of hospital stay, between RC and LC [6]. Furthermore, the high costs associated with RC, including the initial investment in robotic systems, ongoing maintenance, and longer operative times, raise concerns about its cost-effectiveness [7]. Studies have consistently shown that RC is more expensive than LC, which remains a significant barrier to its widespread adoption in clinical practice [7,8].

Given these findings, the overall cost-effectiveness of RC compared to LC remains questionable. While RC may provide certain intraoperative benefits, the substantial additional costs may not be justified, particularly in settings where healthcare resources are limited or where LC already provides satisfactory outcomes [8,9]. This underscores the need for a comprehensive evaluation of both clinical outcomes and cost-effectiveness to determine the viability of RC as a standard procedure for cholecystectomy.

This systematic review aims to evaluate and compare the postoperative outcomes (operative time, bile leak, and postoperative complications) and cost-effectiveness between da Vinci RC and LC. By synthesizing the available evidence, this review aims to provide a comprehensive assessment to guide surgeons and healthcare providers in making informed decisions regarding the choice of surgical technique for cholecystectomy. This evaluation will help determine whether the potential benefits of RC justify its higher costs and whether it should be considered a viable alternative to LC as the standard procedure for gallbladder removal.

## Review

This systematic review and meta-analysis study was carried out according to the Preferred Reporting Items for Systematic Reviews and Meta-Analyses guidelines [10].

Data sources and search strategy

**Figure 1:**
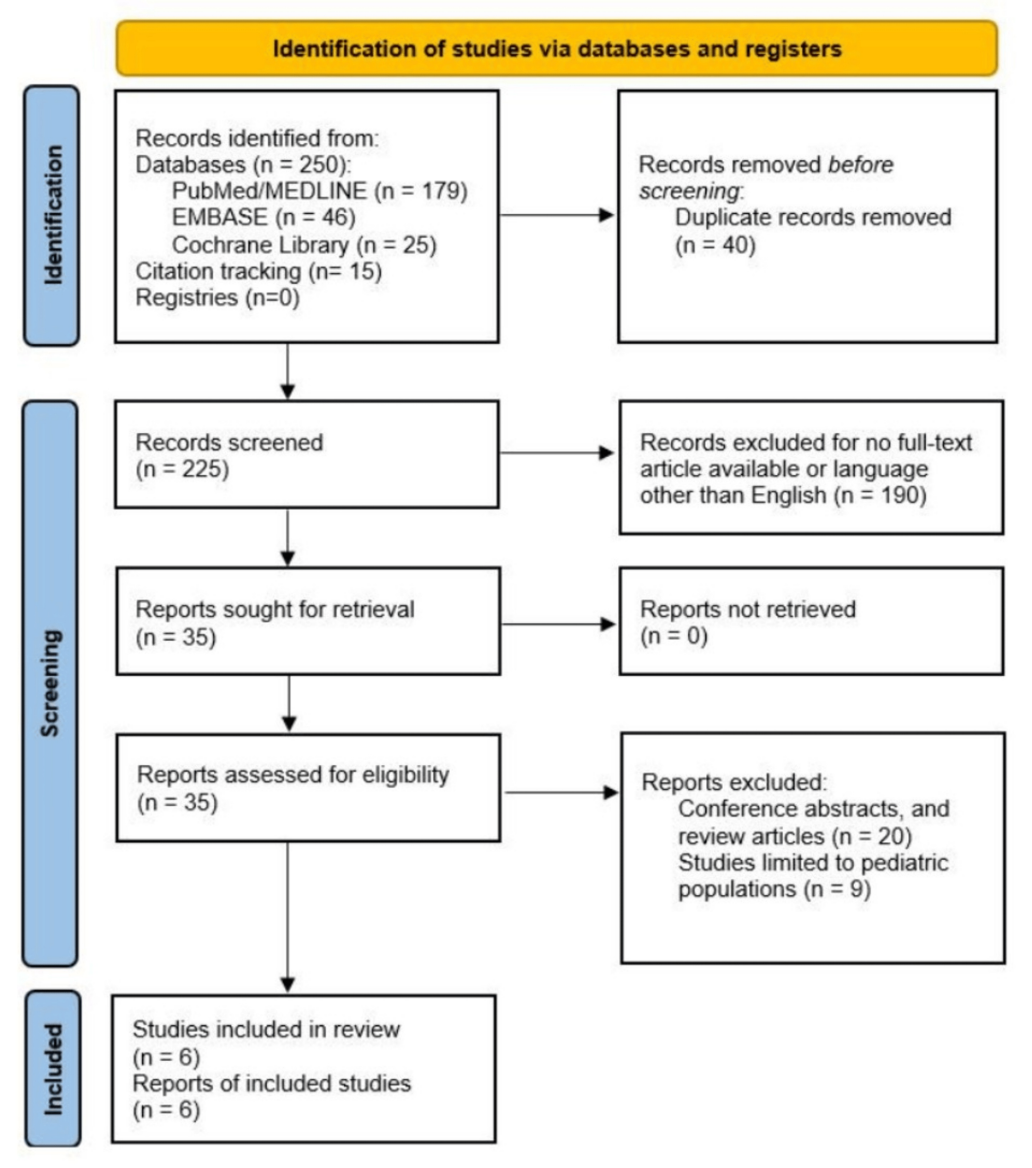
PRISMA flowchart showing data extraction PRISMA: Preferred Reporting Items for Systematic Reviews and Meta-Analyses Source: [13]

A comprehensive literature search was conducted using four major electronic databases: PubMed/MEDLINE, EMBASE, and Cochrane Library. The search strategy was meticulously designed to capture all relevant studies comparing postoperative outcomes and cost-effectiveness between da Vinci RC and LC (Table 1). Medical Subject Headings terms and Boolean operators were used to ensure inclusivity and relevance, allowing for a nuanced and refined search. Key terms included combinations of "Da Vinci robotic cholecystectomy", "laparoscopic cholecystectomy", "postoperative outcomes", and "cost-effectiveness". The search was restricted to studies involving human subjects, published in English, and appearing in peer-reviewed journals. Additional studies were identified by manually screening the references of the initially selected trials to capture any relevant research that might have been missed in the database searches. This snowball approach helped identify key studies that were not indexed under the specific search terms but were nonetheless pertinent to the review [11,12]. Duplicate studies were removed, and the final selection of articles was based on a detailed screening of titles, abstracts, and full texts to confirm relevance to the review objectives. Results of the literature search are represented in Figure 1, following the Preferred Reporting Items for Systematic Reviews and Meta-Analyses 2020 flow diagram.

**Table 1:** PICOT framework

Abbreviation	PICOT inquiries	Description
P	Population	Adult populations
I	Intervention	Da Vinci robotic cholecystectomy
C	Comparator	Laparoscopic cholecystectomy
O	Outcomes	Operative time, bile leak, postoperative complications, and cost-effectiveness
T	Timeframe	Short and long terms

Objectives

The primary objective of this systematic review and meta-analysis is the postoperative outcomes of da Vinci RC and LC surgical procedures (operative time, bile leak, and postoperative complications). The secondary objective is the cost-effectiveness of da Vinci RC and LC surgical procedures.

Inclusion and exclusion criteria

The inclusion criteria focused on comparative trials that evaluated and compared the postoperative outcomes (operative time, bile leak, and postoperative complications) and cost-effectiveness between da Vinci RC and LC. Studies were included if they were conducted in adult populations (age ≥18 years). Both randomized controlled trials (RCTs) and observational comparative studies were considered eligible to capture a wide spectrum of evidence [14]. Exclusion criteria included studies limited to pediatric populations, studies with no full-text or not published in English, conference abstracts, and review articles. Additionally, in cases where multiple publications from the same cohort or overlapping cohorts were identified, the most comprehensive and/or recent study was selected to avoid data duplication. This approach ensured that the data utilized in the review were robust and minimized potential biases from duplicated reporting [15].

Data collection and risk-of-bias assessment

Data extraction was conducted independently by two researchers using a standardized data extraction sheet to ensure consistency and reduce the risk of errors. The data extraction sheet was designed to capture key study characteristics, including sample size, study design, geographic location, patient demographics, details of the surgical procedures, outcomes measured, and results. Following the initial data extraction, the datasets from the independent researchers were compared, and any discrepancies were discussed and resolved through consensus or by consulting a third reviewer. This rigorous verification process was essential in maintaining the integrity and accuracy of the data used in the meta-analysis. The quality of the included studies was assessed using the Newcastle-Ottawa Scale for cohort studies and version 2 of the Cochrane risk-of-bias tool 2 for RCTs [15,16]. Studies were graded as high, medium, or low quality based on their design, sample size, blinding, and outcome reporting, which helped in assessing the reliability of the findings from each study.

Statistical analysis

Statistical analyses were performed using the RevMan 5.4 software (The Cochrane Collaboration, London, UK). For continuous variables, such as operative time, mean differences were calculated to compare the performance of RC and LC. Dichotomous outcomes, such as the incidence of bile leaks and postoperative complications, were analyzed using odds ratios. A random-effect model was applied to account for variability between the studies, given the expected heterogeneity in patient populations, surgical techniques, and healthcare settings [15,16]. Heterogeneity between studies was quantified using the I² statistic, which describes the percentage of variability in effect estimates that is due to heterogeneity rather than sampling error. Cochran's Q test was used to test for the presence of heterogeneity. High I² values (>75%) indicated substantial heterogeneity, prompting further investigation into potential sources, such as differences in study design or geographic variation in healthcare delivery [17,18].

Results

Study Characteristics and Demographics

The review included six trials involving 1,013 patients, comprising three RCTs and three comparative observational studies. These trials were conducted in a variety of healthcare settings across Switzerland, Taiwan, the USA, and Italy, offering a diverse representation of clinical practices and patient populations. This geographic diversity was important for understanding how the findings might be applied across different healthcare systems with varying cost structures and surgical protocols [4]. The included studies varied in sample sizes and surgical approaches, which contributed to the heterogeneity observed in the meta-analysis. Most studies focused on common postoperative outcomes, such as operative time, complication rates, and cost comparisons, allowing for a comprehensive assessment of the comparative effectiveness of Da Vinci RC versus LC. The diverse settings and study designs provide a robust dataset to evaluate the applicability of findings in different healthcare contexts [4].

Risk-of-Bias Assessment

Overall, the three cohort studies were assessed to be of good quality and scored seven stars. Table 2 summarizes the quality assessment scores for the cohort studies. The reasons for not receiving a full quality score for the selection section were as follows: 1) the sample was not truly or somewhat representative of the average in the target population and 2) the outcomes of interest were not demonstrated to be present at the start of the study.

**Table 2:** Quality assessment scores for the cohort studies

Study	Selection (A)	Comparability (B)	Outcome (C)	Overall rating (quality)
Hagen et al. [7]	A1, A2, A3, A4	B1	C1, C2, C3	7 (good)
Li et al. [6]	A1, A2, A3, A4	B1	C1, C2, C3	7 (good)
Su et al. [19]	A1, A2, A3, A4	B1	C1, C2, C3	7 (good)

**Figure 2:**
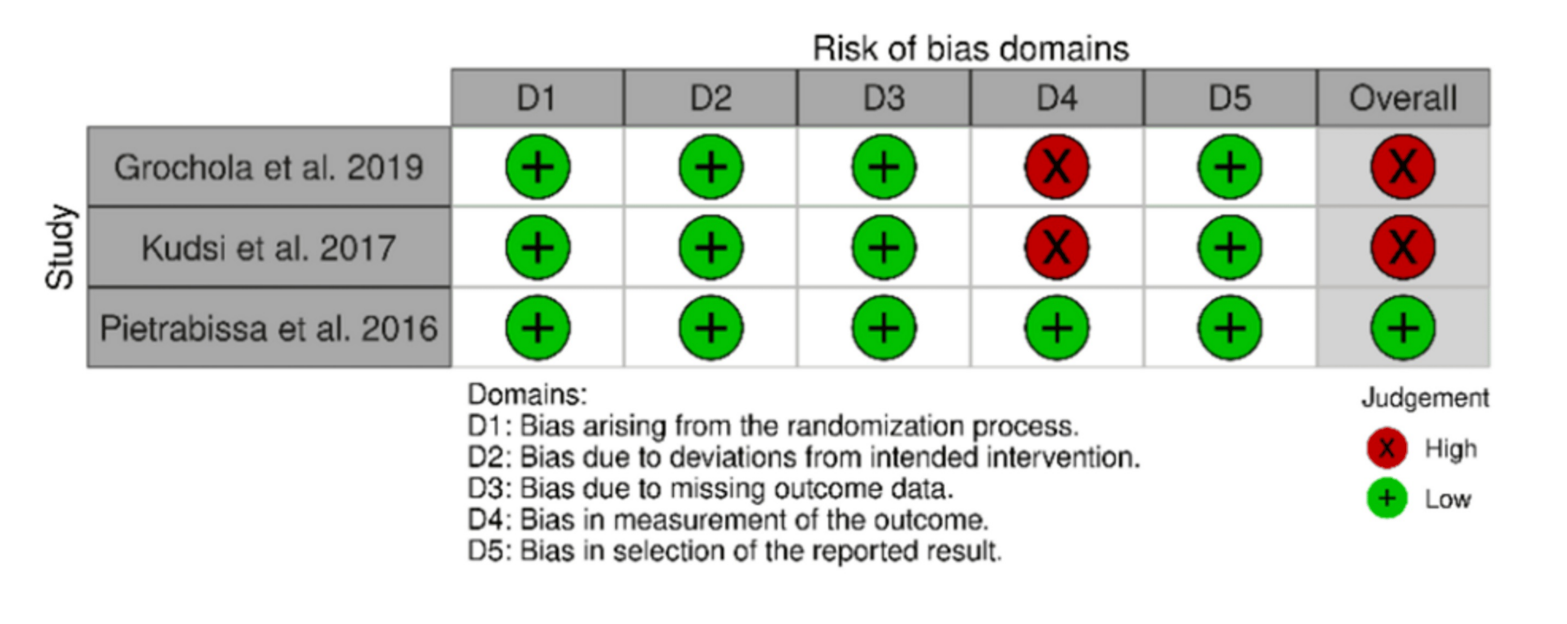
Risk-of-bias domains Source: [20]

Two studies were regarded as having a high risk of bias. This risk of bias (Figure 2) was associated with the measurement of the outcome because it was likely to be influenced by knowledge of the intervention received by study participants (the studies were not double-blinded).

Postoperative Outcomes

**Figure 3:**
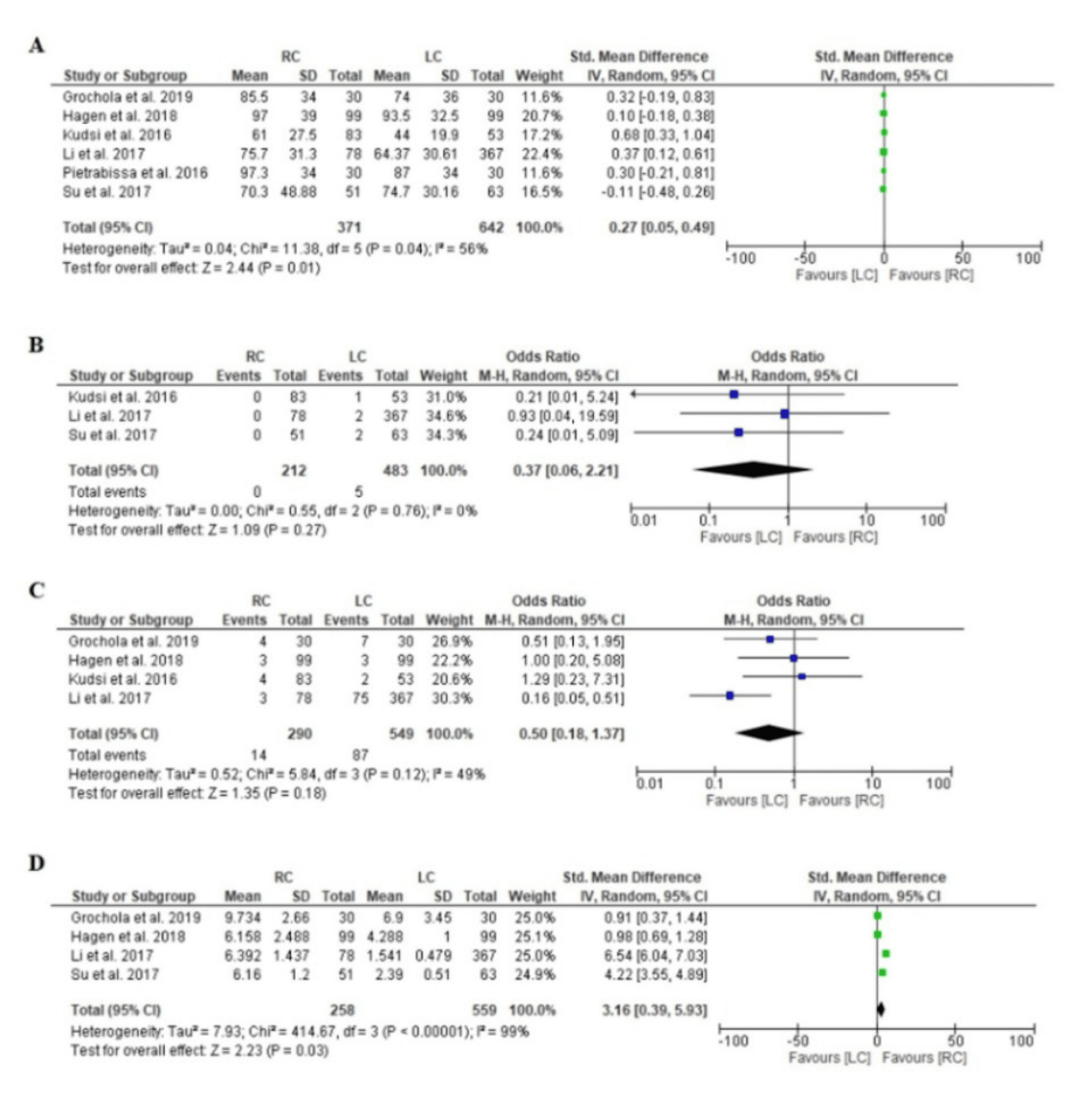
Forest plot for the meta-analysis. (A) SMD in outcomes between the RC and LC groups across multiple studies. The pooled effect size and heterogeneity statistics are included, showing a favor toward the LC group with a slight effect size. (B) OR comparing adverse event occurrences between the RC and LC groups. It indicates no significant difference in adverse events, with a summary effect size leaning slightly toward the LC group but remaining nonsignificant. (C) Mortality ORs between RC and LC. It highlights that mortality differences across studies are not statistically significant, though the overall trend slightly favors LC. (D) SMD for postprocedure recovery outcomes, comparing the RC and LC groups. The overall analysis shows a significant result in favor of LC, suggesting faster recovery outcomes for LC RC: robotic cholecystectomy; LC: laparoscopic cholecystectomy; SD: standard deviation; IV: intravenous; CI: confidence interval; M-H: Mantel-Haenszel; SMD: standard mean difference; OR: odds ratio Source: [21]

Duration of operation: LC was associated with a significantly shorter operative time than RC, with a standardized mean difference of 0.27 minutes (95% CI, 0.05-0.49, p = 0.01). This finding underscores the efficiency of LC over RC in terms of surgical duration, which is a critical factor in operative resource utilization and patient throughput in surgical suites. A significant heterogeneity was detected (χ^2^, 11.38; I^2^, 56%; p = 0.04) (Figure 3A).

Bile leak: The analysis revealed no significant difference in the rates of bile leaks between RC and LC (odds ratio, 0.37; 95% CI, 0.06-2.21; p = 0.27), suggesting comparable safety profiles for both surgical approaches in terms of this complication. No significant heterogeneity was detected (χ2, 0.55; I^2^, 0%; p = 0.76) (Figure 3B).

Postoperative complications: The rates of overall postoperative complications were also similar between the two techniques (odds ratio, 0.50; 95% CI, 0.18-1.37; p = 0.18), indicating that the advanced technological features of RC do not necessarily confer an advantage in reducing complication rates compared to LC. No significant heterogeneity was detected (χ^2^, 5.84; I^2^, 49%; p = 0.12). (Figure 3C).

Cost-effectiveness: RC was found to be significantly more cost-effective than LC, with a standardized mean difference of 3.16 (95% CI, 0.39-5.93, p = 0.03). The high heterogeneity observed (χ^2^, 414.67; I^2^, 99%; p < 0.00001) reflects significant variability in how costs are assessed across different studies, including variations in healthcare infrastructure, robotic equipment costs, and operative efficiency (Figure 3D). This variability underscores the importance of context-specific cost evaluations when considering the adoption of robotic systems in clinical practice. The findings from this systematic review highlight the need for larger, well-conducted multicenter RCTs to validate the current evidence, particularly focusing on standardizing cost assessments and including broader patient populations to improve the generalizability of the results.

Discussion

This meta-analysis provides a comprehensive comparison of LC and da Vinci RC, revealing that LC is associated with shorter operative times and greater cost-effectiveness. These findings are consistent with previous literature, which has frequently highlighted the operational efficiency and economic advantages of LC over RC [4,7]. Despite the sophisticated technological features of RC, such as enhanced precision, 3D visualization, and greater dexterity, the high associated costs raise questions about its widespread adoption, especially in routine surgical practice [2]. The significant financial burden of RC includes the costs of robotic systems, ongoing maintenance, and the requirement for specialized training for surgeons and support staff, all of which contribute to higher overall expenses than LC [7].

Comparison of Findings Across Studies

The included studies uniformly reported that LC offers a shorter operative time than RC. This finding was consistently observed across multiple studies [4,6], reinforcing that LC remains the more time-efficient procedure. Furthermore, the cost analysis demonstrated that RC incurs higher costs than LC [5,7]. These higher costs are attributed not only to the robotic equipment and its maintenance but also to the longer duration of surgeries in some cases, which can lead to increased utilization of operating room resources [7]. Despite the potential for RC to enhance surgical precision and reduce operator fatigue, the meta-analysis did not find significant differences in bile leak rates or postoperative complications between RC and LC [5]. This suggests that the clinical outcomes of RC do not significantly surpass those of LC despite the advanced technology involved [8]. This lack of significant clinical superiority is consistent with prior comparative studies [9], indicating that the incremental technological advancements of RC may not justify its higher costs in terms of improved patient outcomes.

Strengths and Limitations of the Included Studies

Sample size and statistical power: Some of the included studies had relatively small sample sizes, which can limit the statistical power to detect differences in less common outcomes, such as specific complications [6,8]. This limitation suggests that the current evidence base may not be fully equipped to detect subtle differences in clinical outcomes between RC and LC.

Heterogeneity in cost assessments: A significant challenge observed in the meta-analysis was the high heterogeneity in cost assessments across studies (χ^2^, 414.67; I^2^, 99%). This heterogeneity likely reflects differences in healthcare systems, cost structures, and the extent of robotic equipment utilization, which can vary widely between countries and even between hospitals within the same country [7]. Such variability complicates the interpretation of cost-effectiveness data and suggests that localized factors play a substantial role in the financial viability of RC.

Study quality variability: The quality of the studies varied, with cohort studies being rated as good quality and two RCTs presenting a high risk of bias [4,7]. Some studies had limitations in their design, such as inadequate blinding (single-blinded studies) or variability in the experience level of surgeons. These issues can introduce bias and limit the robustness of the findings.

Single-center versus multicenter designs: Most studies were conducted in single centers, which can limit the generalizability of the findings [4,6,7]. Single-center studies may reflect the specific practices and patient populations of those institutions, which may not be representative of broader clinical practice. The inclusion of multicenter data in one study [8] provides a more generalized view but highlights the need for more such studies to validate these findings across diverse settings.

Future Considerations

To better evaluate the comparative effectiveness and cost-efficiency of LC versus RC, several future research directions should be pursued.

Larger, multicenter RCTs: There is a need for larger RCTs conducted across multiple centers to enhance the generalizability of the findings. These trials should include diverse patient populations and settings to ensure that the results apply to a wide range of clinical environments. Such studies could also stratify results based on patient characteristics, such as obesity or comorbidities, to identify subgroups that may benefit more from RC.

Standardization of cost analysis: Future studies should strive to standardize cost analysis methodologies to reduce heterogeneity and provide clearer insights into the economic implications of RC versus LC. This includes comprehensive cost accounting that covers direct, indirect, and long-term costs, such as those related to training, equipment depreciation, and postoperative care.

Evaluation of long-term outcomes and quality of life: Research should also focus on long-term outcomes, including patient satisfaction, quality of life, and the incidence of long-term complications or recurrent gallstone disease. These factors are critical for a holistic evaluation of the benefits of RC and LC, extending beyond the immediate perioperative period.

Impact of technological advancements and surgeon experience: As robotic technology continues to evolve, it will be important to assess whether newer models or improvements in robotic systems can offer better cost-effectiveness or clinical outcomes. Additionally, the impact of surgeon experience and the learning curve associated with RC should be a focus of future studies, as these factors can significantly influence outcomes such as operative time and complication rates.

Cost-effectiveness in different healthcare settings: Further research should explore the cost-effectiveness of RC in different healthcare systems, including low- and middle-income countries, where the financial impact of adopting advanced technologies may be more pronounced. Studies should consider the scalability of RC in resource-limited settings and the potential for cost-saving innovations, such as modular robotic systems or shared robotic platforms.

## Conclusions

This systematic review concludes that da Vinci RC does not offer significant clinical advantages over LC in terms of postoperative outcomes and is associated with higher costs. LC remains the more cost-effective option, reinforcing its status as the preferred approach for cholecystectomy. Future research should focus on conducting larger, multicenter RCTs to confirm these findings and explore the potential role of RC in specific patient subgroups or complex cases.
